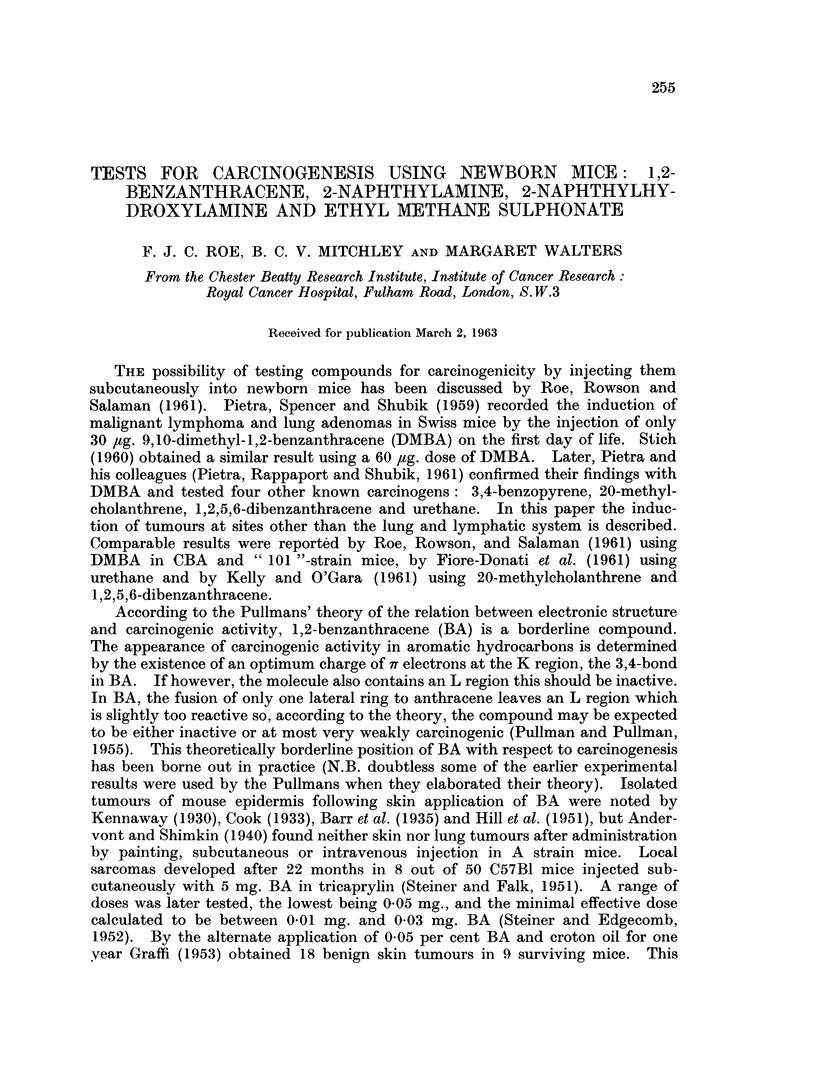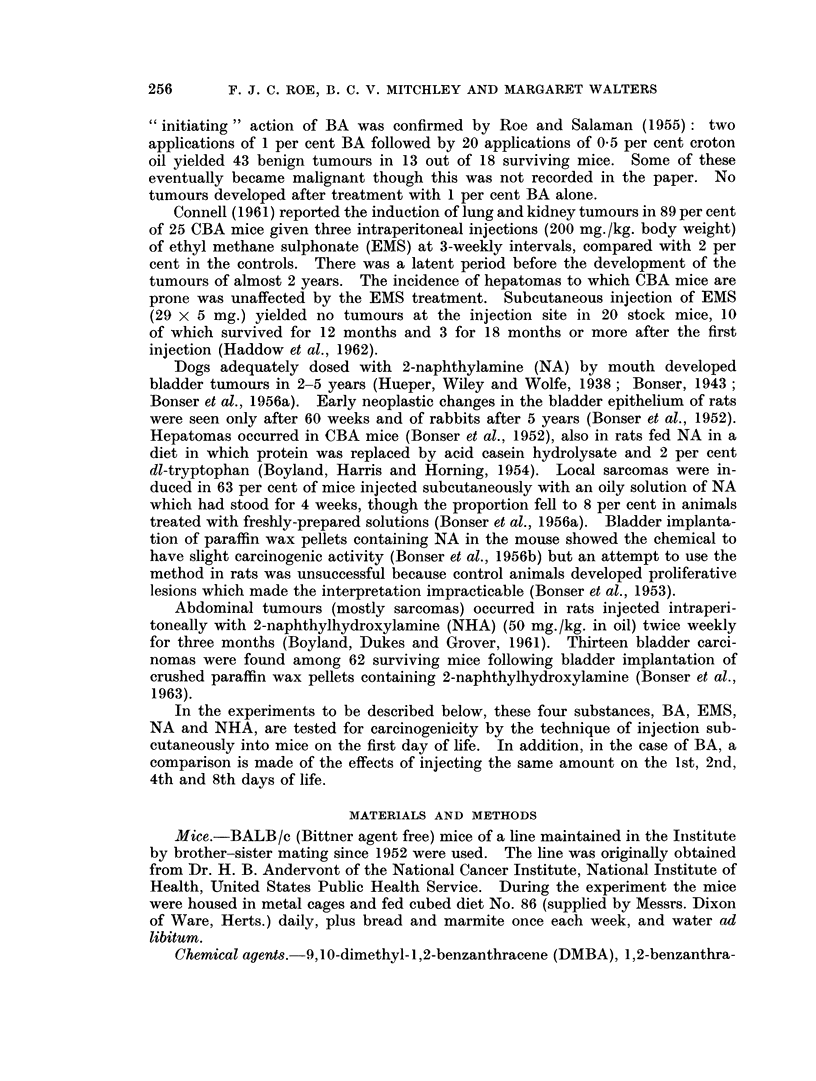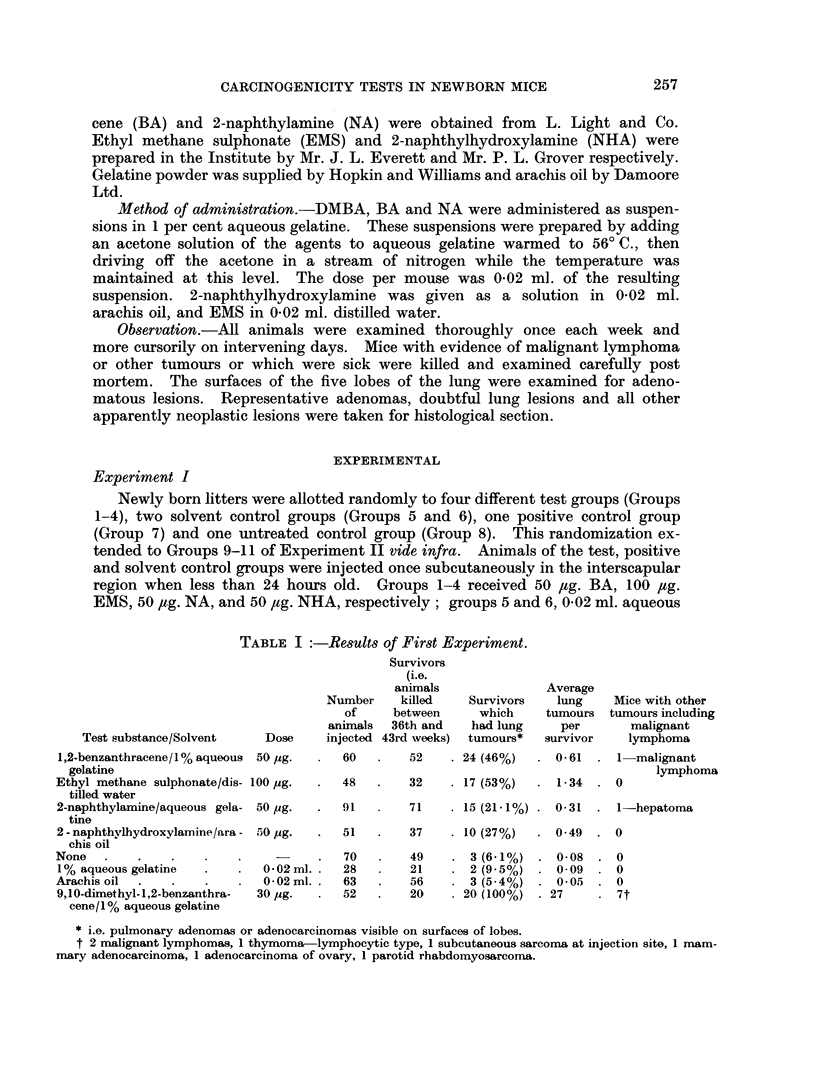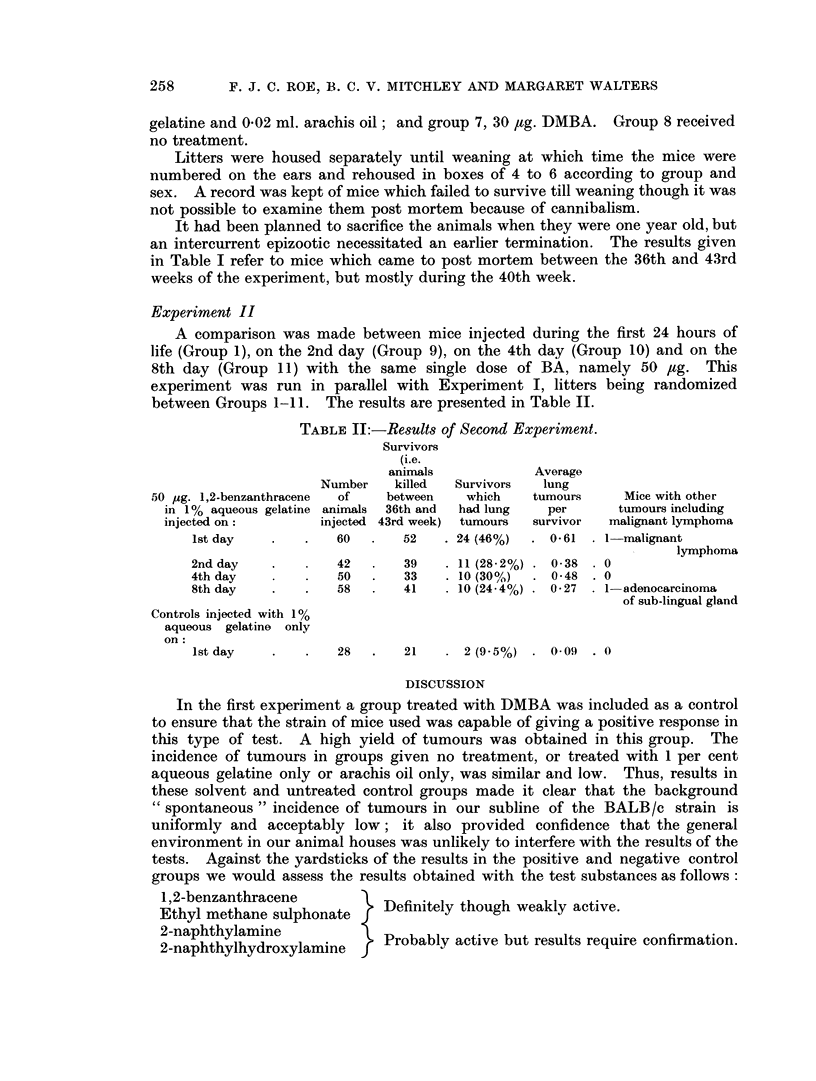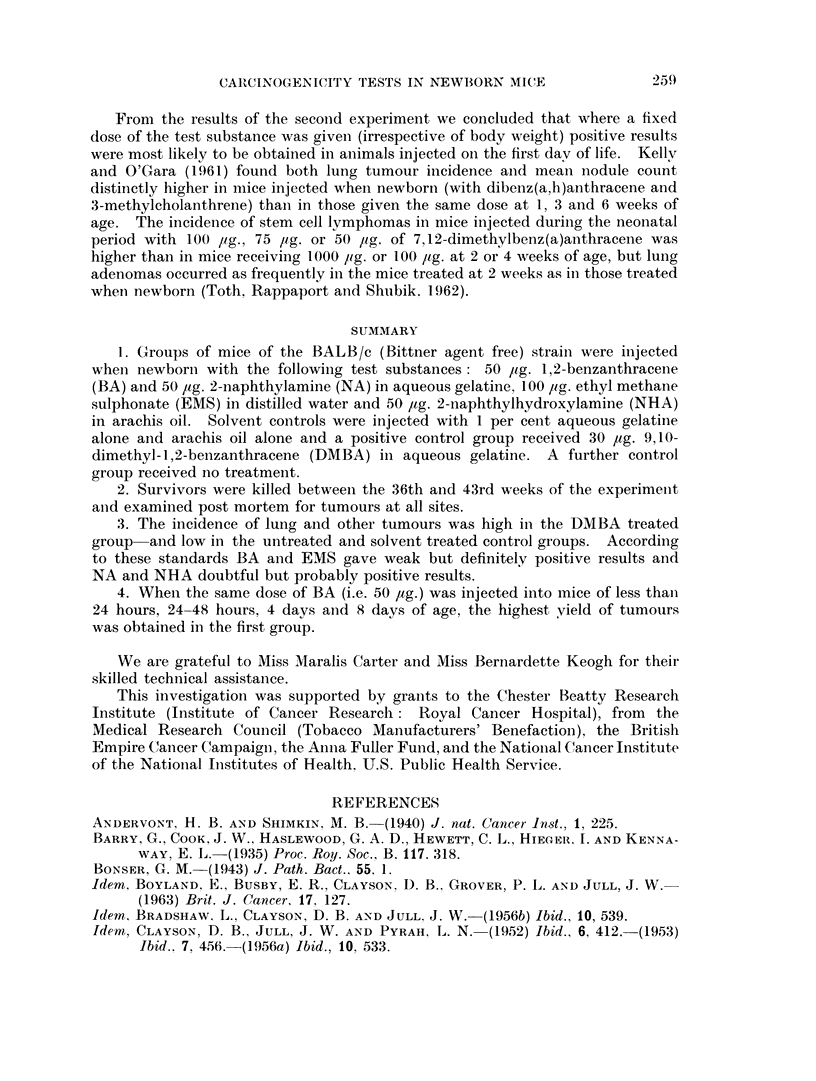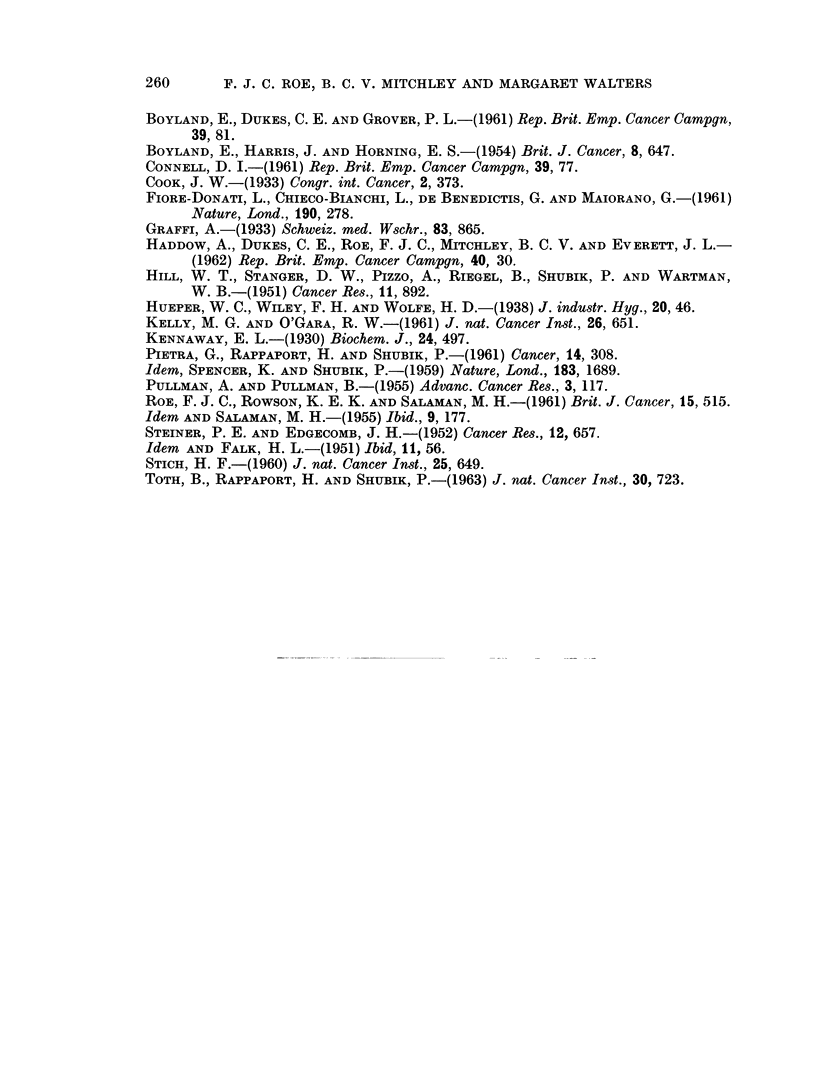# Tests for Carcinogenesis Using Newborn Mice: 1,2-Benzanthracene 2-Naphthylamine, 2-Naphthylhydroxylamine and Ethyl Methane Sulphonate

**DOI:** 10.1038/bjc.1963.36

**Published:** 1963-06

**Authors:** F. J. C. Roe, B. C. V. Mitchley, Margaret Walters


### TESTS FOR CARCINOGENESIS USING NEWBORN MICE: 1,2-BENZANTHRACENE, 2-NAPHTHYLAMINE, 2-NAPHTHYLHYDROXYLAMINE AND ETHYL METHANE SULPHONATE

F. J. C. ROE, B. C. V. MITCHLEY AND MARGARET WALTERS

*From the Chester Beatty Research Institute, Institute of Cancer Research: Royal Cancer Hospital, Fulham Road, London, S.W.3*

Received for publication March 2, 1963

THE possibility of testing compounds for carcinogenicity by injecting them subcutaneously into newborn mice has been discussed by Roe, Rowson and Salaman (1961). Pietra, Spencer and Shubik (1959) recorded the induction of malignant lymphoma and lung adenomas in Swiss mice by the injection of only 30 $\mu$g. 9,10-dimethyl-1,2-benzanthracene (DMBA) on the first day of life. Stich (1960) obtained a similar result using a 60 $\mu$g. dose of DMBA. Later, Pietra and his colleagues (Pietra, Rappaport and Shubik, 1961) confirmed their findings with DMBA and tested four other known carcinogens: 3,4-benzopyrene, 20-methylcholanthrene, 1,2,5,6-dibenzanthracene and urethane. In this paper the induction of tumours at sites other than the lung and lymphatic system is described. Comparable results were reported by Roe, Rowson, and Salaman (1961) using DMBA in CBA and "101"-strain mice, by Fiore-Donati *et al.* (1961) using urethane and by Kelly and O'Gara (1961) using 20-methylcholanthrene and 1,2,5,6-dibenzanthracene.

According to the Pullmans' theory of the relation between electronic structure and carcinogenic activity, 1,2-benzanthracene (BA) is a borderline compound. The appearance of carcinogenic activity in aromatic hydrocarbons is determined by the existence of an optimum charge of $\pi$ electrons at the K region, the 3,4-bond in BA. If however, the molecule also contains an L region this should be inactive. In BA, the fusion of only one lateral ring to anthracene leaves an L region which is slightly too reactive so, according to the theory, the compound may be expected to be either inactive or at most very weakly carcinogenic (Pullman and Pullman, 1955). This theoretically borderline position of BA with respect to carcinogenesis has been borne out in practice (N.B. doubtless some of the earlier experimental results were used by the Pullmans when they elaborated their theory). Isolated tumours of mouse epidermis following skin application of BA were noted by Kennaway (1930), Cook (1933), Barr *et al.* (1935) and Hill *et al.* (1951), but Andervont and Shimkin (1940) found neither skin nor lung tumours after administration by painting, subcutaneous or intravenous injection in A strain mice. Local sarcomas developed after 22 months in 8 out of 50 C57Bl mice injected subcutaneously with 5 mg. BA in tricaprylin (Steiner and Falk, 1951). A range of doses was later tested, the lowest being 0·05 mg., and the minimal effective dose calculated to be between 0·01 mg. and 0·03 mg. BA (Steiner and Edgecomb, 1952). By the alternate application of 0·05 per cent BA and croton oil for one year Graffi (1953) obtained 18 benign skin tumours in 9 surviving mice. This

"initiating" action of BA was confirmed by Roe and Salaman (1955): two applications of 1 per cent BA followed by 20 applications of 0·5 per cent croton oil yielded 43 benign tumours in 13 out of 18 surviving mice. Some of these eventually became malignant though this was not recorded in the paper. No tumours developed after treatment with 1 per cent BA alone.

Connell (1961) reported the induction of lung and kidney tumours in 89 per cent of 25 CBA mice given three intraperitoneal injections (200 mg./kg. body weight) of ethyl methane sulphonate (EMS) at 3-weekly intervals, compared with 2 per cent in the controls. There was a latent period before the development of the tumours of almost 2 years. The incidence of hepatomas to which CBA mice are prone was unaffected by the EMS treatment. Subcutaneous injection of EMS ($29 \times 5$ mg.) yielded no tumours at the injection site in 20 stock mice, 10 of which survived for 12 months and 3 for 18 months or more after the first injection (Haddow *et al.*, 1962).

Dogs adequately dosed with 2-naphthylamine (NA) by mouth developed bladder tumours in 2–5 years (Hueper, Wiley and Wolfe, 1938; Bonser, 1943; Bonser *et al.*, 1956a). Early neoplastic changes in the bladder epithelium of rats were seen only after 60 weeks and of rabbits after 5 years (Bonser *et al.*, 1952). Hepatomas occurred in CBA mice (Bonser *et al.*, 1952), also in rats fed NA in a diet in which protein was replaced by acid casein hydrolysate and 2 per cent *dl*-tryptophan (Boyland, Harris and Horning, 1954). Local sarcomas were induced in 63 per cent of mice injected subcutaneously with an oily solution of NA which had stood for 4 weeks, though the proportion fell to 8 per cent in animals treated with freshly-prepared solutions (Bonser *et al.*, 1956a). Bladder implantation of paraffin wax pellets containing NA in the mouse showed the chemical to have slight carcinogenic activity (Bonser *et al.*, 1956b) but an attempt to use the method in rats was unsuccessful because control animals developed proliferative lesions which made the interpretation impracticable (Bonser *et al.*, 1953).

Abdominal tumours (mostly sarcomas) occurred in rats injected intraperitoneally with 2-naphthylhydroxylamine (NHA) (50 mg./kg. in oil) twice weekly for three months (Boyland, Dukes and Grover, 1961). Thirteen bladder carcinomas were found among 62 surviving mice following bladder implantation of crushed paraffin wax pellets containing 2-naphthylhydroxylamine (Bonser *et al.*, 1963).

In the experiments to be described below, these four substances, BA, EMS, NA and NHA, are tested for carcinogenicity by the technique of injection subcutaneously into mice on the first day of life. In addition, in the case of BA, a comparison is made of the effects of injecting the same amount on the 1st, 2nd, 4th and 8th days of life.

### MATERIALS AND METHODS

*Mice.*—BALB/c (Bittner agent free) mice of a line maintained in the Institute by brother–sister mating since 1952 were used. The line was originally obtained from Dr. H. B. Andervont of the National Cancer Institute, National Institute of Health, United States Public Health Service. During the experiment the mice were housed in metal cages and fed cubed diet No. 86 (supplied by Messrs. Dixon of Ware, Herts.) daily, plus bread and marmite once each week, and water *ad libitum.*

*Chemical agents.*—9,10-dimethyl-1,2-benzanthracene (DMBA), 1,2-benzanthracene (BA) and 2-naphthylamine (NA) were obtained from L. Light and Co. Ethyl methane sulphonate (EMS) and 2-naphthylhydroxylamine (NHA) were prepared in the Institute by Mr. J. L. Everett and Mr. P. L. Grover respectively. Gelatine powder was supplied by Hopkin and Williams and arachis oil by Damoore Ltd.

*Method of administration.*—DMBA, BA and NA were administered as suspensions in 1 per cent aqueous gelatine. These suspensions were prepared by adding an acetone solution of the agents to aqueous gelatine warmed to 56° C., then driving off the acetone in a stream of nitrogen while the temperature was maintained at this level. The dose per mouse was 0·02 ml. of the resulting suspension. 2-naphthylhydroxylamine was given as a solution in 0·02 ml. arachis oil, and EMS in 0·02 ml. distilled water.

*Observation.*—All animals were examined thoroughly once each week and more cursorily on intervening days. Mice with evidence of malignant lymphoma or other tumours or which were sick were killed and examined carefully post mortem. The surfaces of the five lobes of the lung were examined for adenomatous lesions. Representative adenomas, doubtful lung lesions and all other apparently neoplastic lesions were taken for histological section.

### EXPERIMENTAL

### *Experiment I*

Newly born litters were allotted randomly to four different test groups (Groups 1–4), two solvent control groups (Groups 5 and 6), one positive control group (Group 7) and one untreated control group (Group 8). This randomization extended to Groups 9–11 of Experiment II *vide infra.* Animals of the test, positive and solvent control groups were injected once subcutaneously in the interscapular region when less than 24 hours old. Groups 1–4 received 50 μg. BA, 100 μg. EMS, 50 μg. NA, and 50 μg. NHA, respectively ; groups 5 and 6, 0·02 ml. aqueous

TABLE I :—*Results of First Experiment.*

| Test substance/Solvent | Dose | Number of animals injected | Survivors (i.e. animals killed between 36th and 43rd weeks) | Survivors which had lung tumours* | Average lung tumours per survivor | Mice with other tumours including malignant lymphoma |
|---|---|---|---|---|---|---|
| 1,2-benzanthracene/1% aqueous gelatine | 50 μg. | 60 | 52 | 24 (46%) | 0·61 | 1—malignant lymphoma |
| Ethyl methane sulphonate/distilled water | 100 μg. | 48 | 32 | 17 (53%) | 1·34 | 0 |
| 2-naphthylamine/aqueous gelatine | 50 μg. | 91 | 71 | 15 (21·1%) | 0·31 | 1—hepatoma |
| 2-naphthylhydroxylamine/arachis oil | 50 μg. | 51 | 37 | 10 (27%) | 0·49 | 0 |
| None | — | 70 | 49 | 3 (6·1%) | 0·08 | 0 |
| 1% aqueous gelatine | 0·02 ml. | 28 | 21 | 2 (9·5%) | 0·09 | 0 |
| Arachis oil | 0·02 ml. | 63 | 56 | 3 (5·4%) | 0·05 | 0 |
| 9,10-dimethyl-1,2-benzanthracene/1% aqueous gelatine | 30 μg. | 52 | 20 | 20 (100%) | 27 | 7† |

* i.e. pulmonary adenomas or adenocarcinomas visible on surfaces of lobes.

† 2 malignant lymphomas, 1 thymoma—lymphocytic type, 1 subcutaneous sarcoma at injection site, 1 mammary adenocarcinoma, 1 adenocarcinoma of ovary, 1 parotid rhabdomyosarcoma.

gelatine and 0·02 ml. arachis oil ; and group 7, 30 μg. DMBA. Group 8 received no treatment.

Litters were housed separately until weaning at which time the mice were numbered on the ears and rehoused in boxes of 4 to 6 according to group and sex. A record was kept of mice which failed to survive till weaning though it was not possible to examine them post mortem because of cannibalism.

It had been planned to sacrifice the animals when they were one year old, but an intercurrent epizootic necessitated an earlier termination. The results given in Table I refer to mice which came to post mortem between the 36th and 43rd weeks of the experiment, but mostly during the 40th week.

*Experiment II*

A comparison was made between mice injected during the first 24 hours of life (Group 1), on the 2nd day (Group 9), on the 4th day (Group 10) and on the 8th day (Group 11) with the same single dose of BA, namely 50 μg. This experiment was run in parallel with Experiment I, litters being randomized between Groups 1–11. The results are presented in Table II.

TABLE II:—*Results of Second Experiment.*

| 50 μg. 1,2-benzanthracene in 1% aqueous gelatine injected on : | Number of animals injected | Survivors (i.e. animals killed between 36th and 43rd week) | Survivors which had lung tumours | Average lung tumours per survivor | Mice with other tumours including malignant lymphoma |
|---|---|---|---|---|---|
| 1st day | 60 | 52 | 24 (46%) | 0·61 | 1—malignant lymphoma |
| 2nd day | 42 | 39 | 11 (28·2%) | 0·38 | 0 |
| 4th day | 50 | 33 | 10 (30%) | 0·48 | 0 |
| 8th day | 58 | 41 | 10 (24·4%) | 0·27 | 1—adenocarcinoma of sub-lingual gland |
| Controls injected with 1% aqueous gelatine only on : | | | | | |
| 1st day | 28 | 21 | 2 (9·5%) | 0·09 | 0 |

DISCUSSION

In the first experiment a group treated with DMBA was included as a control to ensure that the strain of mice used was capable of giving a positive response in this type of test. A high yield of tumours was obtained in this group. The incidence of tumours in groups given no treatment, or treated with 1 per cent aqueous gelatine only or arachis oil only, was similar and low. Thus, results in these solvent and untreated control groups made it clear that the background "spontaneous" incidence of tumours in our subline of the BALB/c strain is uniformly and acceptably low ; it also provided confidence that the general environment in our animal houses was unlikely to interfere with the results of the tests. Against the yardsticks of the results in the positive and negative control groups we would assess the results obtained with the test substances as follows :

| | |
|---|---|
| 1,2-benzanthracene<br>Ethyl methane sulphonate | Definitely though weakly active. |
| 2-naphthylamine<br>2-naphthylhydroxylamine | Probably active but results require confirmation. |

From the results of the second experiment we concluded that where a fixed dose of the test substance was given (irrespective of body weight) positive results were most likely to be obtained in animals injected on the first day of life. Kelly and O'Gara (1961) found both lung tumour incidence and mean nodule count distinctly higher in mice injected when newborn (with dibenz(a,h)anthracene and 3-methylcholanthrene) than in those given the same dose at 1, 3 and 6 weeks of age. The incidence of stem cell lymphomas in mice injected during the neonatal period with 100 $\mu$g., 75 $\mu$g. or 50 $\mu$g. of 7,12-dimethylbenz(a)anthracene was higher than in mice receiving 1000 $\mu$g. or 100 $\mu$g. at 2 or 4 weeks of age, but lung adenomas occurred as frequently in the mice treated at 2 weeks as in those treated when newborn (Toth, Rappaport and Shubik, 1962).

SUMMARY

1. Groups of mice of the BALB/c (Bittner agent free) strain were injected when newborn with the following test substances: 50 $\mu$g. 1,2-benzanthracene (BA) and 50 $\mu$g. 2-naphthylamine (NA) in aqueous gelatine, 100 $\mu$g. ethyl methane sulphonate (EMS) in distilled water and 50 $\mu$g. 2-naphthylhydroxylamine (NHA) in arachis oil. Solvent controls were injected with 1 per cent aqueous gelatine alone and arachis oil alone and a positive control group received 30 $\mu$g. 9,10-dimethyl-1,2-benzanthracene (DMBA) in aqueous gelatine. A further control group received no treatment.

2. Survivors were killed between the 36th and 43rd weeks of the experiment and examined post mortem for tumours at all sites.

3. The incidence of lung and other tumours was high in the DMBA treated group—and low in the untreated and solvent treated control groups. According to these standards BA and EMS gave weak but definitely positive results and NA and NHA doubtful but probably positive results.

4. When the same dose of BA (i.e. 50 $\mu$g.) was injected into mice of less than 24 hours, 24–48 hours, 4 days and 8 days of age, the highest yield of tumours was obtained in the first group.

We are grateful to Miss Maralis Carter and Miss Bernardette Keogh for their skilled technical assistance.

This investigation was supported by grants to the Chester Beatty Research Institute (Institute of Cancer Research: Royal Cancer Hospital), from the Medical Research Council (Tobacco Manufacturers' Benefaction), the British Empire Cancer Campaign, the Anna Fuller Fund, and the National Cancer Institute of the National Institutes of Health, U.S. Public Health Service.

### REFERENCES

ANDERVONT, H. B. AND SHIMKIN, M. B.—(1940) *J. nat. Cancer Inst.*, **1**, 225.

BARRY, G., COOK, J. W., HASLEWOOD, G. A. D., HEWETT, C. L., HIEGER, I. AND KENNAWAY, E. L.—(1935) *Proc. Roy. Soc.*, B. **117**, 318.

BONSER, G. M.—(1943) *J. Path. Bact.*, **55**, 1.

*Idem*, BOYLAND, E., BUSBY, E. R., CLAYSON, D. B., GROVER, P. L. AND JULL, J. W.—(1963) *Brit. J. Cancer*, **17**, 127.

*Idem*, BRADSHAW, L., CLAYSON, D. B. AND JULL, J. W.—(1956*b*) *Ibid.*, **10**, 539.

*Idem*, CLAYSON, D. B., JULL, J. W. AND PYRAH, L. N.—(1952) *Ibid.*, **6**, 412.—(1953) *Ibid.*, **7**, 456.—(1956*a*) *Ibid.*, **10**, 533.

BOYLAND, E., DUKES, C. E. AND GROVER, P. L.—(1961) *Rep. Brit. Emp. Cancer Campgn*, **39**, 81.
BOYLAND, E., HARRIS, J. AND HORNING, E. S.—(1954) *Brit. J. Cancer*, **8**, 647.
CONNELL, D. I.—(1961) *Rep. Brit. Emp. Cancer Campgn*, **39**, 77.
COOK, J. W.—(1933) *Congr. int. Cancer*, **2**, 373.
FIORE-DONATI, L., CHIECO-BIANCHI, L., DE BENEDICTIS, G. AND MAIORANO, G.—(1961) *Nature, Lond.*, **190**, 278.
GRAFFI, A.—(1933) *Schweiz. med. Wschr.*, **83**, 865.
HADDOW, A., DUKES, C. E., ROE, F. J. C., MITCHLEY, B. C. V. AND EVERETT, J. L.—(1962) *Rep. Brit. Emp. Cancer Campgn*, **40**, 30.
HILL, W. T., STANGER, D. W., PIZZO, A., RIEGEL, B., SHUBIK, P. AND WARTMAN, W. B.—(1951) *Cancer Res.*, **11**, 892.
HUEPER, W. C., WILEY, F. H. AND WOLFE, H. D.—(1938) *J. industr. Hyg.*, **20**, 46.
KELLY, M. G. AND O'GARA, R. W.—(1961) *J. nat. Cancer Inst.*, **26**, 651.
KENNAWAY, E. L.—(1930) *Biochem. J.*, **24**, 497.
PIETRA, G., RAPPAPORT, H. AND SHUBIK, P.—(1961) *Cancer*, **14**, 308.
*Idem*, SPENCER, K. AND SHUBIK, P.—(1959) *Nature, Lond.*, **183**, 1689.
PULLMAN, A. AND PULLMAN, B.—(1955) *Advanc. Cancer Res.*, **3**, 117.
ROE, F. J. C., ROWSON, K. E. K. AND SALAMAN, M. H.—(1961) *Brit. J. Cancer*, **15**, 515.
*Idem* AND SALAMAN, M. H.—(1955) *Ibid.*, **9**, 177.
STEINER, P. E. AND EDGECOMB, J. H.—(1952) *Cancer Res.*, **12**, 657.
*Idem* AND FALK, H. L.—(1951) *Ibid*, **11**, 56.
STICH, H. F.—(1960) *J. nat. Cancer Inst.*, **25**, 649.
TOTH, B., RAPPAPORT, H. AND SHUBIK, P.—(1963) *J. nat. Cancer Inst.*, **30**, 723.